# Nationwide distribution of varicella-zoster virus clades in China

**DOI:** 10.1186/s12879-016-1863-x

**Published:** 2016-10-07

**Authors:** Songtao Xu, Mukai Chen, Huanying Zheng, Haiyan Wang, Meng Chen, Jianhui Zhou, Wang Shuang, Pengbo Yu, Chaofeng Ma, Jilan He, Daxing Feng, Zhu Zhen, Zhang Yan, Mao Naiying, Aili Cui, Qiuhua Wu, Mengyuan Qi, Chongshan Li, Xiaoguang Xu, Wenbo Xu

**Affiliations:** 1 National Institute for Viral Disease Control and Prevention, China Center for Disease Control and Prevention, Beijing City, 102206 China; 2 The First Affiliated Hospital of Sun Yat-sen University, Guangzhou City, 510080 Guangdong Province China; 3 Guangdong Center for Disease Control and Prevention, Guangzhou City, 510300 Guangdong Province China; 4 Shandong Center for Disease Control and Prevention, Jinan City, 250014 Shandong Province China; 5 Beijing Center for Disease Control and Prevention, Beijing City, 100021 China; 6 Jilin province Center for Disease Control and Prevention, Changchun City, 130021 Jilin Province China; 7 Shaanxi Center for Disease Control and Prevention, Xian City, 710012 Shannxi Province China; 8 Xi’an city Center for Disease Control and Prevention, Xian City, 710031 Shannxi Province China; 9 Sichuan Center for Disease Control and Prevention, Chengdu City, 610014 Sichuan Province China; 10 Henan Center for Disease Control and Prevention, Zhengzhou City, 450016 Henan Province China; 11 Department of Neurosurgery, Second Affiliated Hospital of Dalian Medical University, Dalian, 116027 Liaoning Province China; 12 Shanghai Center for Disease Control and Prevention, Shanghai City, 200336 China

**Keywords:** Varicella-zoster virus, Clade, Varicella vaccine adverse event, Genetic profile, China

## Abstract

**Background:**

In 2010, a universal nomenclature for varicella-zoster virus (VZV) clades was established, which is very useful in the monitoring of viral evolution, recombination, spread and genetic diversity. Currently, information about VZV clades has been disclosed worldwide, however, there are limited data regarding the characterization of circulating VZV clades in China, even where varicella remains widely epidemic.

**Methods:**

From 2008 to 2012, clinical samples with varicella or zoster were collected in General Hospital in eight provinces and analyzed by PCR, restriction endonuclease digestion and sequencing. The viral clades were determined by analysis of five single nucleotide polymorphisms (SNPs) within the 447-bp fragment of open reading frame (ORF) 22, and the restriction fragment length polymorphisms (RFLPs) of ORF 38 (*Pst*I), ORF 54 (*Bgl*I) and ORF 62 (*Sma*I) were evaluated to understand genetic diversity of VZV and determinate varicella vaccine adverse event (VVAE).

**Results:**

Seventy-seven varicella and 11 zoster samples were identified as being positive for VZV. The five SNPs profile showed that the majority of VZV strains belonged to clade 2, but clade 5 and clade 4 strains were also found in Guangdong. The RFLPs analysis of the DNA fragments of ORF 38, 54 and 62 showed that 85 of these samples were characterized as *Pst*I + *Bgl*I + *Sam*I-, and the remaining three VZV strains from varicella patients were characterized as *Pst*I-*Bgl*I + *Sam*I+ which is the genetic profile of VVAEs.

**Conclusions:**

The study suggested that the predominant clade 2 VZVs had been continually circulating since at least the 1950s in China. Nearly all VZV strains except VVAEs possessed the genetic profile of *Pst*I + *Bgl*I + *Sam*-. However, the other clades were also found to be co-circulating with clade 2, especially in the border regions. These results highlighted the need for the constant and broad use of virologic surveillance to provide an important genetic baseline for varicella control and vaccination programs in China.

## Background

Varicella-zoster virus (VZV) is highly contagious and causes two diseases. The primary infection results in varicella (chickenpox), which usually occurs early in life. Subsequently, the virus establishes a lifelong latent infection in the sensory nerve ganglia, which reactivates to cause zoster (shingles) under conditions of declining immunity that are most commonly associated age [1].

Genotyping of VZV contributes to reveal viral diversity, recombination, evolution patterns and transmission pathways. To achieve these goals, several groups have published different schemes by using molecular techniques such as the sequencing, restriction fragment length polymorphisms (RFLPs) and single nucleotide polymorphisms (SNPs) since the 1990s, however, the different nomenclature methods will not facilitate interchange of information [2–7]. In 2010, Breuer et al. summarized the previous nomenclature methods and proposed a novel nomenclature for VZV: clade 1–5 and two putative clades (VI and VII). The new universal nomenclature will be useful for the interchange and comparison of genotyping data worldwide [6, 8, 9].

The universal childhood varicella vaccination program was initiated in the United States (US) in 1995 [10, 11]. Currently, although varicella vaccines are licensed and available throughout the world, the varicella vaccination has been recommended as a universal childhood immunization in only a small number of countries including Canada, Germany, Australia, Korea and Japan, where high coverage rates have been attained, and vaccination has resulted in a significant decline in varicella-related incidence, morbidity and mortality [12, 13]. Due to the absence of varicella vaccination programs in most countries, varicella remains a worldwide epidemic and was estimated that the global varicella disease burden includes 4.2 million severe cases and 4200 deaths annually [14]. In China, the varicella vaccine is not included in the national immunization program, which results in poor varicella control and epidemic conditions, but it’s available for purchase on private basis for vaccination of children. However, application of the live-attenuated varicella vaccine may cause varicella vaccine adverse events (VVAE). Surveillance of the VVAE plays a key role in evaluating side effects of the vaccine and helps physicians in appropriate decision making.

This study describes the clade distribution and genetic profile of circulating VZV strains throughout China, and distinguishes vaccine strains from wild-type strains. These data provide important genetic background that will facilitate efforts for controlling varicella and zoster in China in the future.

## Methods

### Ethics statement

This study was approved by the Ethics Committee of National Institute for Viral Disease Control and Prevention, China Center for Disease Control and Prevention. Individual written informed consent was obtained from the parents or guardians of all participants.

### Clinical samples

**Fig. 1:**
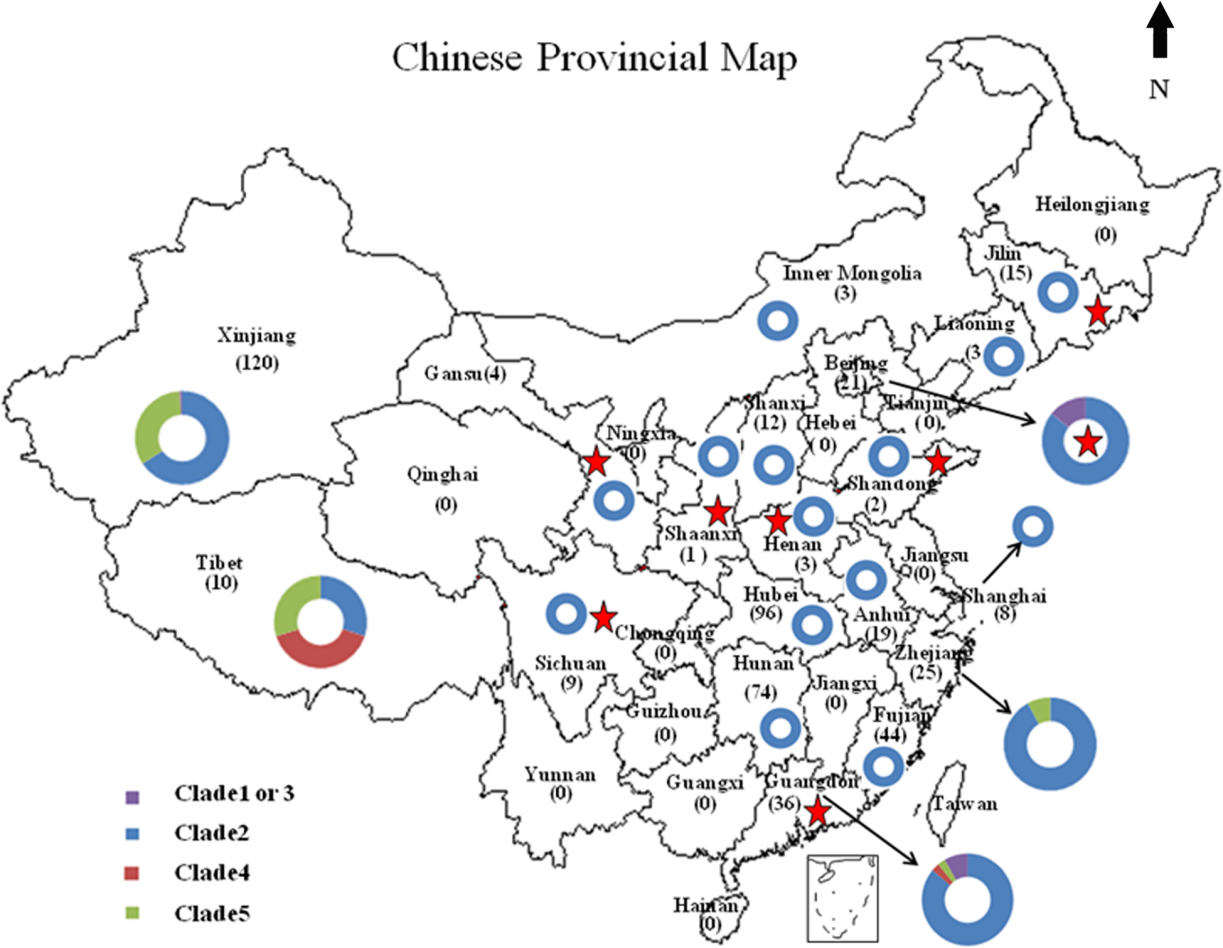
Distribution of VZV clades in China. 1. Numbers in parenthesis indicate the number of varicella and/or zoster strains; 2. The eight provinces described in this study are labelled with an asterisk

From 2008 to 2012, the suspected varicella or zoster samples were collected from outpatients who were clinically diagnosed with varicella or zoster in General Hospitals. These patients stayed residentially in eight provinces (Beijing, Jilin, Guangdong, Henan, Shaanxi, Gansu, Sichuan, and Shandong) located in eastern, southern, western, northern and central of mainland China. These provinces cross subtropical (Guangdong) and temperate climates (the other provinces) and should be sufficiently representative (Fig. 1).

### Viral DNA extraction and polymerase chain reaction (PCR)

Viral DNA was extracted from 200 μl vesicle fluid or throat swab samples using the QIAamp DNA Mini Kit (Qiagen, Valencia, CA). To characterize viral clades and distinguish vaccine strains from wide-type strains, the PCR amplification of ORF 22, 38, 54 and 62 were performed in accordance with previously described protocols [15–18]. One Chinese vaccine strain and one wide type strain were used for the positive controls, the DNase-free water was used for the negative control. The 447-bp region of ORF 22 (Strain Dumas, Genbank No. X04370) encompassing five SNPs (37902, 38019, 38055, 38081, and 38177) was sufficient to characterize between viral clades [2, 6]. However, discrimination between clades 1 and 3 required the addition of a sixth SNP (39394) to differentiate between the two clades. The genetic markers of VZV that are most commonly considered in vaccine adverse events and epidemiological studies include the RFLPs of ORFs 38 (*Pst*I), 54 (*Bgl*I) and 22 (*Sma*I) [16, 19]. The PCR assays were performed using the PCR amplification kit (Takara, Dalian, China) according to the manufacturer’s instruction. The template amplifications were performed using a GeneAmp 9700 thermocycler (Applied Biosystems, Grand Island, NY, USA). The PCR products were purified using a QIAquick Gel Extraction Kit (Qiagen) and evaluated by electrophoretic separation on 2 % agarose gels.

### Sequencing and restriction enzyme reactions

The 447-bp region of ORF 22 were sequenced using an ABI PRISM 3100 genetic analyzer (Applied Biosystems, Hitachi, Japan) according to the manufacturer’s instruction, and the sequence data were assembled and edited using Sequencher software version 4.0.5 (GeneCodes, Ann Arbor, MI). The five SNPs were analyzed for VZV clades identification using BioEdit version 7.0 (Tom Hall, North Carolina State University, Raleigh, NC). Restriction endonuclease digestion of the PCR products (ORF38, ORF54 and ORF62) was performed with 8 μl of the PCR products, 15 U endonuclease (*Pst*I, *Bgl*I and *Sma*I) (Takara, Dalian, China), and 2 μl of the accompanying 10× endonuclease buffer. The final reaction volume was adjusted to 20 μl with DNase- and RNase-free water. The analysis of RFLPs was performed according to the protocols previously described in the literature [2, 16].

## Results

### Case confirmation

From 2008 to 2012, a total of 126 clinical samples were collected in eight provinces, including 115 throat swabs from 115 varicella patients and 11 vesicular fluids from elven zoster patients. All patients were born and spent their childhoods in China. No information was available about the VZV exposure history including varicella vaccination, except for five patients with mild varicella within 3 weeks after varicella vaccination. The ages of 115 patients with varicella ranged between 3 and 25 years (mean age 7.5 years); the ages of 11 patients with zoster ranged between 40 and 68 years (mean age 50 years).

A total 88 of 126 samples were tested VZV positive using the PCR screening of ORF 22 (447-bp), including 77 throat swabs from varicella patients and all 11 vesicular fluids from zoster patients. The throat swabs from three of the five varicella patients with mild varicella after varicella vaccination were also positive.

### Viral clade determination

The 447-bp fragment (ORF 22) of 88 VZV samples was successfully sequenced, the five SNPs profile (37902: G, 38019: G, 38055: C, 38081: C and 38177: A) disclosed that most of endemic VZV strains were attributed to clade 2; however, one clade 5 (37902: A, 38019: G, 38055: T, 38081: C and 38177: G) strain from a 7-year-old child and one clade 4 (37902: A, 38019: A, 38055: C, 38081: C and 38177: A) strain from a 64-year-old elder with zoster were found in Guangdong in 2009. The analysis of the sixth SNP (in the position 39394) was not performed due to the lack of clade 1 or 3 strains (Table 1).

**Table 1:** Genetic profile of eighty-eight VZV strains in eight provinces

Province	Collection time	No. of V or Z	Clade	Residue at ORF 22 position					ORF38 (Pstl)	ORF54 (Bgll)	ORF62 (Small)
				37902	38019	38055	38081	38177			
Guangdong	2008–2010	V(21), Z(10)	2	G	G	C	C	A	+	+	-
Guangdong	2009	V (1)	4	A	A	C	C	A	+	+	-
Guangdong	2009	Z (1)	5	A	G	T	C	G	+	+	-
Sichuan	2009	V (9)	2	G	G	C	C	A	+	+	-
Beijing	2009	V (18)	2	G	G	C	C	A	+	+	-
Henan	2010	V (1)	2	G	G	C	C	A	+	+	-
Henan	2010	V (2^a^)	2	G	G	C	C	A	-	+	+
Shaanxi	2012	V (1^a^)	2	G	G	C	C	A	-	+	+
Shandong	2012	V(2)	2	G	G	C	C	A	+	+	-
Gansu	2012	V (7)	2	G	G	C	C	A	+	+	-
Jilin	2011–2012	V (15)	2	G	G	C	C	A	+	+	-

1. *V* varicella; *Z* herpes zoster

2. All genome positions are based on the published sequence for the VZV reference strain: Dumas (Clade 1; Accession # XO4370)

3. Numbers in parenthesis indicate the number of varicella and/or zoster strains

4. ^a^in right corner indicates the VVAEs

### Distinguishing vaccine strains from wild-type of VZV strains

For these positive VZV strains, three PCR amplifications of ORF 38 (350-bp), 54 (222-bp) and 62 (268-bp) were performed to distinguish vaccine strains from wild-type strains. The three target bands were obtained for each of the samples and digested with *Pst*I, *Bgl*I and *Sma*I enzymes, respectively. The 350-bp amplification products were cleaved to the 250 bp and 100 bp fragments by *Pst*I in most of the samples, except for three samples that were collected from the Henan and Shaanxi provinces. The 222-bp amplification products were cleaved to 137 bp and 85 bp by *Bgl*I in all of the samples. The two genetic markers that were characterized in the most VZV strains possessed the *Pst*I + *Bgl*I+ pattern, with the exception of 3 samples (*Pst*I-*Bgl*I+). For the 268-bp fragment, most VZV samples were cleaved to 153-bp, 79-bp, and 36 bp (*Sma*I-) except the 3 strains that were cleaved to 112-bp, 79-bp, 41-bp and 36 bp (*Sma*I+). The results of the analysis of the three genetic makers indicated that most of the VZVs belonged to wild-type strains, whereas the three VZV strains from patients within 3 weeks after varicella vaccination possessed the genetic profile (*Pst*I-*Bgl*I + *Sma*I+) of the Oka varicella vaccine (Table 1).

## Discussion

Information about the molecular epidemiology of VZV has been proven very useful for characterizing endemic strains, tracing transmission pathways, and distinguishing vaccine strains from wild-type strains. In 2010, Breuer proposed a novel nomenclature for VZV: clade 1–5 and two putative clades (VI and VII) that were identified by Roman numerals until confirmed to be a clade. The new universal nomenclature will be useful for the interchange and comparison of genotyping data worldwide [6]. According to the universal nomenclature, the worldwide distribution of VZV clades has been established. Clade 1 and clade 3 represent the dominate clades of the circulating VZV in Europe and the Americas, where the clade 4 and 5 strains are frequently identified due to the immigration of people with African origin. The clade VI strains were also found in France (10 %) and Italy (11 %), and one might speculate that clade VI strains are more common than clade 3 strains in Southern Europe [20]. To date, only one clade VII strain was exclusively isolated in the US in 2002 [21]; in Asia, VZV clade 2 has been the dominant clade according to previous publications from Korea, Japan, and China; however, clade 2 strains were not found circulating in India, Nepal and Bangladesh, where clade 4 and 5 strains were dominant [2, 22–24].

Several previous publications (mostly in Chinese) have disclosed information about the genotypes and genetic profiles of VZV, but all of them focused on one province or prefecture and were limited [2, 23, 25–32]. Overall, 420 VZV strains collected from 115 varicella patients and 305 zoster patients were characterized, which were detected during the period from 2000 to 2015 in 13 provinces (Table 2). In this study, 88 VZV strains collected from eight provinces where no VZV investigation was reported were characterized (Table 1). With the incorporation of previously published data, this study contributes towards the knowledge of the molecular epidemiology profile of prevalent VZVs circulating in mainland China.

**Table 2:** Clade summary of the VZV strains isolated in mainland China according to previous publications

Province	Collection time	No. of V or Z	Clade	References
Guangdong	before 2000	V (3)	4	[2]
Beijing	before 2000	V (3)	1 or 3	[2]
Shanghai	2007	V (8)	2	[25, 26]
Hubei	2008	V (16), Z (80)	2	[32]
Anhui	2007–2008	V (2), Z (17)	2	[23]
Liaoning	2009	V (3)	2	[33]
Inner Mongolia	2010	V (3)	2	[28]
Zhejiang	2009–2011	V (25)	2 (23), 5 (2)	[37]
Fujian	2010–2011	V (10), Z (34)	2	[41]
Tibet	2011	V (6), Z (4)	5 (3), 4 (4), 2 (3)	[27]
Xinjiang	2012–2013	V (4), Z (116)	2 (79), 5 (40), 1 or 3 (1)	[30]
Shanxi	2013	V (12)	2	[31]
Hunan	2013–2015	V (20), Z (54)	2	[29]

1. *V* varicella; *Z* herpes zoster

2. Numbers in parenthesis indicate the number of varicella and/or zoster strains

In northern China (Jilin, Liaoning, Beijing, Shanxi and Inner Mongolian provinces), all clinical samples from varicella patients since 2009 belonged to clade 2 [28, 31, 33]. However, three clade 1 or 3 VZV strains circulating before 2000 were identified in Beijing by Loparev [2]. We speculate that the three clade 1 or 3 strains, which were either imported or random cases, were not the predominate clades in light of the current studies. In western China, 120 VZV strains including four varicella and 116 zoster samples were described in Xinjiang. The results showed that four varicella strains belonged to clade 2 and 116 zoster strains were distributed into 3 clades: 75 in clade 2, 40 in clade 5, and one in clade 1 or 3. Geography might cause multiple clade strains to co-circulate because it borders Gansu province, where clade 2 strains predominate. Additionally, Southern Xinjiang borders Pakistan and India, where clade 5 strains have been circulating [34, 35]. Northern Xinjiang borders Russia, where the clade 1 and 3 predominate [36]. The genetic profiles of the RFLPs were characterized as 120 *Pst*I+, 119 *BgI*I+, one *BgI*I- and 120 *Sma*I-, which indicated that the genetic profile of most strains was *Pst*I + *BgI*I+, except for one *Pst*I + *BgI*I- strain which is the genetic profile of strains from Europe and North America [30]. In Tibet, ten positive VZV strains were identified and belonged to three clades, three in clade 2, three in clade 1 or 3, and four in clade 5, which is probably due to the special geographical location, as it borders Pakistan, India and Nepal, where clades 1 or 3 and clade 5 predominate [2]. However, information about VZV clades is currently unavailable for Yunan which is also a very special location because it borders Myanmar, Vietnam and Laos where the characterization of the VZV clade has never been reported. The strengthening of the virologic surveillance is critical in Yunan in the near future. In southern China (Guangdong, Hunan and Hubei provinces), a total of 206 positive VZV strains, those isolated from 141 varicella and 65 zoster cases, were identified. All of them belonged to clade 2, except for one, which belonged to clade 5, and one that belonged to clade 4 and was found in Guangdong. The clade 5 strain was isolated from a 7-year-old varicella patient. The clade 4 strain was isolated from a 63-year-old zoster patient. Loparev et al. also published that three clade 4 strains circulating before the year 2000 were found in Guangdong [2]. These results suggest that the minority clade 4 and 5 strains might have been circulating in Guangdong for a long time. This situation might result from frequent international communication, especially among the hundreds of thousands of Africans residing in Guangdong. However, this should be further validated by studies with large sample numbers in the future. In eastern China (Shandong, Anhui, Shanghai, Zhejiang, and Fujian provinces), the clade characterization had been already highlighted, and all VZV strains from varicella or zoster patients belonged to clade 2 except two imported clade 5 strains based on the epidemiological survey in Zhejiang (Tables 1 and 2) (Fig. 1) [37, 38].

Primary infection with VZV usually occurs during childhood, at approximately 10 years of age [39]. Previously published data have shown that the strains of virus causing zoster in a patient are identical to the primary varicella strain that infected that patient [5]. The current study shows that although other clades of VZV strains were found to co-circulate in the border regions such as Xinjiang, Tibet and Guangdong, the majority of VZV strains from zoster patients belonged to clade 2. The oldest patient was 68 years old, which indicated that the clade 2 VZV strains had been continually circulating in China since at least the 1950s.

Since the varicella vaccine is a live attenuated vaccine, varicella-like rash after vaccination is rare but can occur and is defined as a varicella vaccine adverse event (VVAE), an increasingly common side effect of varicella vaccination. The data of the Vaccine Adverse Event Reporting System of the U.S. showed the mild varicella-like rash would occur in 3 to 5 % of varicella vaccine recipients [5]. The RFLP of a variable region is a useful tool to better understand VZV genetic characterization and determination of varicella or zoster vaccine adverse event. Previously RFLPs analysis showed that the majority of wild-type strains from Europe and North America (temperate climate) were *Pst*I *+ Bgl*I*-*. Wild-type strains in Africa, Asia or the Caribbean (tropical and subtropical climates) were *Pst*I *+ Bgl*I*+*, whereas the Oka vaccine strain (vOka) was *Pst*I*-Bgl*I*+*, and the Japanese Oka-like wild-type strains were *Pst*I*-Bgl*I*+/Pst*I *+ Bgl*I*+*, which indicates that the RFLP analysis of the *Pst*I and *Bgl*I failed to distinguish wild-type strains from Oka vaccine strains [8, 16, 18]. In 2000, RFLP analysis of the *Sma*I in ORF 62 was developed, which had been used to distinguish vaccine strains from the wild-type strains. This marker has been shown to be stable and can be confidently used to identify vaccine strains [16]. However, with the identification of more unique SNPs within the vaccine virus, it may be preferable to sequence the SNPs in order to identify vaccine strains [9]. In this study, three VVAEs from varicella patients in Shaanxi and Henan were identified according to the genetic profile of RFLPs: *Pst*I-*BgI*I + *Sma*I+ [16, 40], which is limited report about the VVAEs in China until now. Although the varicella vaccine is not included in the national immunization program in China, with the improvement of prevention awareness of varicella, more children will be vaccinated, which could result in more VVAEs. Therefore, it is very important to enhance the ability to determine VVAEs that will enable physicians to make informed decisions and to relieve the burden of disease in patients.

## Conclusions

In conclusion, this study confirms that VZV clade 2 represented the dominant clade nationwide and has been continually circulating in China since at least the 1950s. There were other clade strains co-circulating with clade 2 in the border regions. The genetic profile of the 3 RFLPs within ORF 38, 54 and 62 was usually *Pst*I + *BgI*I + *Sma*I-, except for three VVAEs (*Pst*I-*BgI*I + *Sma*I+). This report identified the clade distribution of the circulating VZV strains throughout China and determined the VVAEs. These results emphasize the identification of VVAEs and the importance of a more systematic collection of VZV strains, especially in the border regions, to propose more effective control strategies and to establish an accurate genetic baseline for VZVs in China in the near future.

## Abbreviations

ORF: Open reading frame
RFLP: Restriction fragment length polymorphism
SNP: Single nucleotide polymorphism
VVAE: Varicella vaccine adverse event
VZV: Varicella-zoster virus

## References

[1] Overturf GD (2000). American Academy of Pediatrics. Committee on Infectious Diseases. Technical report: prevention of pneumococcal infections, including the use of pneumococcal conjugate and polysaccharide vaccines and antibiotic prophylaxis. Pediatrics.

[2] Loparev VN, Gonzalez A, Deleon-Carnes M, Tipples G, Fickenscher H, Torfason EG, Schmid DS (2004). Global identification of three major genotypes of varicella-zoster virus: longitudinal clustering and strategies for genotyping. J Virol.

[3] Muir WB, Nichols R, Breuer J (2002). Phylogenetic analysis of varicella-zoster virus: evidence of intercontinental spread of genotypes and recombination. J Virol.

[4] Faga B, Maury W, Bruckner DA, Grose C (2001). Identification and mapping of single nucleotide polymorphisms in the varicella-zoster virus genome. Virology.

[5] Pickering LK e: Red book: report of the Committee on Infectious Diseases.American Academy of Pediatrics 2000, 25th ed. :624–638.

[6] Breuer J, Grose C, Norberg P, Tipples G, Schmid DS (2010). A proposal for a common nomenclature for viral clades that form the species varicella-zoster virus: summary of VZV Nomenclature Meeting 2008, Barts and the London School of Medicine and Dentistry, 24–25 July 2008. J Gen Virol.

[7] Adams SG, Dohner DE, Gelb LD (1989). Restriction fragment differences between the genomes of the Oka varicella vaccine virus and American wild-type varicella-zoster virus. J Med Virol.

[8] Peters GA, Tyler SD, Grose C, Severini A, Gray MJ, Upton C, Tipples GA (2006). A full-genome phylogenetic analysis of varicella-zoster virus reveals a novel origin of replication-based genotyping scheme and evidence of recombination between major circulating clades. J Virol.

[9] Tyler SD, Peters GA, Grose C, Severini A, Gray MJ, Upton C, Tipples GA (2007). Genomic cartography of varicella-zoster virus: a complete genome-based analysis of strain variability with implications for attenuation and phenotypic differences. Virology.

[10] Schmid DS, Jumaan AO (2010). Impact of varicella vaccine on varicella-zoster virus dynamics. Clin Microbiol Rev.

[11] Goldman GS, King PG (2013). Review of the United States universal varicella vaccination program: Herpes zoster incidence rates, cost-effectiveness, and vaccine efficacy based primarily on the Antelope Valley Varicella Active Surveillance Project data. Vaccine.

[12] Lu L, Suo L, Li J, Zhai L, Zheng Q, Pang X, Bialek SR, Wang C (2012). A varicella outbreak in a school with high one-dose vaccination coverage, Beijing, China. Vaccine.

[13] Lu L, Wang C, Suo L, Li J, Liu W, Pang X, Seward JF (2013). Varicella disease in Beijing in the era of voluntary vaccination, 2007 to 2010. Pediatr Infect Dis J.

[14] WHO (2014). Varicella and Herpes Zoster Vaccination Position Paper.

[15] Loparev V, Martro E, Rubtcova E, Rodrigo C, Piette JC, Caumes E, Vernant JP, Schmid DS, Fillet AM (2007). Toward universal varicella-zoster virus (VZV) genotyping: diversity of VZV strains from France and Spain. J Clin Microbiol.

[16] Loparev VN, Argaw T, Krause PR, Takayama M, Schmid DS (2000). Improved identification and differentiation of varicella-zoster virus (VZV) wild-type strains and an attenuated varicella vaccine strain using a VZV open reading frame 62-based PCR. J Clin Microbiol.

[17] Loparev VN, Rubtcova EN, Bostik V, Govil D, Birch CJ, Druce JD, Schmid DS, Croxson MC (2007). Identification of five major and two minor genotypes of varicella-zoster virus strains: a practical two-amplicon approach used to genotype clinical isolates in Australia and New Zealand. J Virol.

[18] LaRussa P, Lungu O, Hardy I, Gershon A, Steinberg SP, Silverstein S (1992). Restriction fragment length polymorphism of polymerase chain reaction products from vaccine and wild-type varicella-zoster virus isolates. J Virol.

[19] Bergval I, Coll F, Schuitema A, de Ronde H, Mallard K, Pain A, McNerney R, Clark TG, Anthony RM (2015). A proportion of mutations fixed in the genomes of in vitro selected isogenic drug-resistant Mycobacterium tuberculosis mutants can be detected as minority variants in the parent culture. FEMS Microbiol Lett.

[20] Loparev VN, Rubtcova EN, Bostik V, Tzaneva V, Sauerbrei A, Robo A, Sattler-Dornbacher E, Hanovcova I, Stepanova V, Splino M (2009). Distribution of varicella-zoster virus (VZV) wild-type genotypes in northern and southern Europe: evidence for high conservation of circulating genotypes. Virology.

[21] Sergeev N, Rubtcova E, Chizikov V, Schmid DS, Loparev VN (2006). New mosaic subgenotype of varicella-zoster virus in the USA: VZV detection and genotyping by oligonucleotide-microarray. J Virol Methods.

[22] Kim KH, Choi YJ, Song KH, Park WB, Jeon JH, Park SW, Kim HB, Kim NJ, Oh MD (2011). Genotype of varicella-zoster virus isolates in South Korea. J Clin Microbiol.

[23] Liu J, Wang M, Gan L, Yang S, Chen J (2009). Genotyping of clinical varicella-zoster virus isolates collected in China. J Clin Microbiol.

[24] Schmidt-Chanasit J, Sauerbrei A (2011). Evolution and world-wide distribution of varicella-zoster virus clades. Infect Genet Evol.

[25] Yang Ji-xing JL-w, et al: Research of genotype of varicella-zoster virus in Shanghai. Chin J Health Lab Technol. 2008, 18:223-225

[26] Li Chongshan LL (2009). Gene characteristics of varicella-zoster virus. Disease Surveillance.

[27] Liu Lan DB-z, et al: Research on the genotypes of clinical varicella-zoster virus isolateds in Tibet. Chin J Microbiol Immunol 2012, 32(11):934-938.

[28] Lu Ai-tao GW-d, et al: Genotype analysis of the pathogen of an outbreak of varicella. Prac Prev Med. 2012; 19:1302-1304

[29] Peng Yan-qian ZW-j, et al: The main genotype of Varicella-zoster virus circulating in Hunan. Chin Foreign Medl Res. 2015; 13(25):142-144.

[30] Qu Yuan-yuan YH, et al: Genotypic analysis of clinical varicella-zoster virus isolations in Xinjiang. Clin J lepr Skin Dis. 2015; 31:526-530

[31] Shi Wei LJ (2015). Genotype analysis of varicella-zoster virus in Yingze of Taiyuan in 2013. J Prac Med Techniques.

[32] Zhou Xiao-yong ZZ-l, et al: Genotype analysis of varicella-zoster virus in Wuhan. Chin J Dermatol. 2009; 42(10):688-690.

[33] Wang yan My, et al.: Genotype analysis of varicella-zoster virus in Liaoning province. Chin J Public Health. 2012; 28(1):76–78.

[34] Kaushik KS, Lahiri KK, Chumber SK, Gupta RM, Kumar S, Kapila K, Karade S (2008). Molecular characterization of clinical varicella-zoster strains from India and differentiation from the oka vaccine strain. Jpn J Infect Dis.

[35] Inoue H, Motani-Saitoh H, Sakurada K, Ikegaya H, Yajima D, Hayakawa M, Sato Y, Otsuka K, Kobayashi K, Nagasawa S (2010). Determination of the geographical origin of unidentified cadavers based on geographical differences in genotype of varicella-zoster virus. J Med Virol.

[36] Springfeld C, Sauerbrei A, Filusch A, Konstandin M, Hartschuh W, Sauer P, Encke J, Stremmel W, Schnitzler P (2009). Fatal varicella in an immunocompromised adult associated with a European genotype E2 variant of varicella zoster virus. J Clin Virol.

[37] Hu Feng-jiao JS-lea: Chickenpox cases monitoring and epidemic strains gene polymorphism analysis in Ningbo,Zhejiang. Inter J Epidemiol Infect Dis. 2013; 40(4):254-257.

[38] Grose C (2012). Pangaea and the Out-of-Africa Model of Varicella-Zoster Virus Evolution and Phylogeography. J Virol.

[39] Gnann JW, Whitley RJ (2002). Clinical practice. Herpes zoster. N Engl J Med.

[40] Hondo R, Yogo Y, Yoshida M, Fujima A, Itoh S (1989). Distribution of varicella-zoster virus strains carrying a PstI-site-less mutation in Japan and DNA change responsible for the mutation. Jpn J Exp Med.

[41] Chen Ting-ting WH, et al: Genotyping of varicella-zoster virus clinical strains isolated in Fujian, China. Chin J Microbiol Immunol. 2012; 32(7):614-617.

